# Dendritic Cell Targeting Effectively Boosts T Cell Responses Elicited by an HIV Multiepitope DNA Vaccine

**DOI:** 10.3389/fimmu.2017.00101

**Published:** 2017-02-07

**Authors:** Juliana de Souza Apostólico, Victória Alves Santos Lunardelli, Marcio Massao Yamamoto, Higo Fernando Santos Souza, Edecio Cunha-Neto, Silvia Beatriz Boscardin, Daniela Santoro Rosa

**Affiliations:** ^1^Department of Microbiology, Immunology and Parasitology, Federal University of São Paulo (UNIFESP/EPM), São Paulo, Brazil; ^2^Institute for Investigation in Immunology (iii), INCT, São Paulo, Brazil; ^3^Department of Parasitology, Institute of Biomedical Sciences, University of São Paulo, São Paulo, Brazil; ^4^Laboratory of Clinical Immunology and Allergy-LIM60, University of São Paulo School of Medicine, São Paulo, Brazil; ^5^Laboratory of Immunology, Heart Institute (InCor), University of São Paulo School of Medicine, São Paulo, Brazil

**Keywords:** HIV, dendritic cells, multiepitope vaccine, CD4^+^ T cell, monoclonal antibody

## Abstract

Despite several efforts in the last decades, an efficacious HIV-1 vaccine is still not available. Different approaches have been evaluated, such as recombinant proteins, viral vectors, DNA vaccines, and, most recently, dendritic cell (DC) targeting. This strategy is based on DC features that place them as central for induction of immunity. Targeting is accomplished by the use of chimeric monoclonal antibodies directed to DC surface receptors fused to the antigen of interest. In this work, we targeted eight promiscuous HIV-derived CD4^+^ T cell epitopes (HIVBr8) to the DEC205^+^ DCs by fusing the multiepitope immunogen to the heavy chain of αDEC205 (αDECHIVBr8), in the presence of the TLR3 agonist poly (I:C). In addition, we tested a DNA vaccine encoding the same epitopes using homologous or heterologous prime-boost regimens. Our results showed that mice immunized with αDECHIVBr8 presented higher CD4^+^ and CD8^+^ T cell responses when compared to mice that received the DNA vaccine (pVAXHIVBr8). In addition, pVAXHIVBr8 priming followed by αDECHIVBr8 boosting induced higher polyfunctional proliferative and cytokine-producing T cell responses to HIV-1 peptides than homologous DNA immunization or heterologous αDEC prime/DNA boost. Based on these results, we conclude that homologous prime-boost and heterologous boosting immunization strategies targeting CD4^+^ epitopes to DCs are effective to improve HIV-specific cellular immune responses when compared to standalone DNA immunization. Moreover, our results indicate that antigen targeting to DC is an efficient strategy to boost immunity against a multiepitope immunogen, especially in the context of DNA vaccination.

## Introduction

Since HIV was discovered in the 1980s, there has been a remarkable progress in the treatment for AIDS. Despite impressive advances in the scientific knowledge and numerous trials, a safe and effective preventive HIV vaccine is still not available. The majority of licensed vaccines provide protection against other pathogens by the induction of neutralizing antibodies (nAbs), but strategies that focused on the development of an effective humoral immunity for HIV have failed so far. The RV144 trial was the only to demonstrate some level of efficacy (~31.2%) against HIV infection by inducing env-specific CD4^+^ T cells as well as antibodies that were able to bind to HIV, but not to neutralize it (1, 2). Moreover, the tested vaccine regimen induced proliferating CD4^+^ T cells with a cytotoxic profile (3). A T cell vaccine able to elicit potent cellular immune responses showed marked protection against simian immunodeficiency (SIV) challenge in non-human primates (4). Janes et al. (5) showed that Gag-specific T cells induced by the Merck Ad5Gag–nef–pol vaccine were associated with reduced viremia after HIV-1 infection.

The role of CD4^+^ T cells to support immunity places them as key for viral clearance, ensuring homeostasis. The important role of CD4 T cell responses during HIV (6) and non-human primate SIV infection is now clear (7). They can provide help for CD8^+^ cytotoxic T cells to control virus replication (8), especially in the mucosal region (9). In addition, HIV-specific CD4^+^ T cell responses promote B cell differentiation leading to generation or maintenance of nAbs in natural infection (10). Furthermore, HIV-specific CD4^+^ T cells can control viral replication by direct cytotoxicity (11) or indirectly through the secretion of soluble antiviral mediators (CCL3, CCL4, and CCL5) (12). A polyfunctional Gag-specific CD4^+^ T cell response was inversely correlated with virus load and directly with the HIV-specific CD8^+^ T cell response in HIV-infected long-term non-progressor individuals (13). Furthermore, expression of specific HLA class II alleles has a considerable impact on the control of HIV replication. HLA-DRB1*15:02 is significantly associated with HIV control (14) and elite controllers that express HLA-DRB1*13 and HLA-DQB1*06 class II HLA molecule showed superior mucosal Gag-specific CD4^+^ T cell responses that produced simultaneously IFNγ, TNFα, and IL-2 when compared to non-controllers or individuals in highly active antiretroviral therapy (HAART) (9). Thus, it is now accepted that an effective vaccine should also promote broad and polyfunctional CD4^+^ T responses against HIV infection (15).

The inclusion of appropriate HIV-1 epitopes recognized by CD4^+^ T cells may thus play an essential role in the induction of immune responses to a HIV vaccine candidate. Our group has previously described a set of conserved HIV-1 CD4^+^ T cell epitopes from the whole proteome of the HIV-1 B subtype consensus that promiscuously bound to multiple HLA-DR, -DQ, and -DP molecules. Peripheral blood mononuclear cells from 90% of HIV-seropositive individuals recognized these epitopes, and the strongest responses were found among long-term non-progressors (16). Epitope-based vaccines focus responses on epitopes with desirable properties and prevent responses to neutral or deleterious epitopes (17). A DNA vaccine encoding the mentioned conserved epitopes (HIVBr18) induced broad specific CD4^+^ and CD8^+^ T responses in transgenic mice expressing human HLA class II alleles (HLA-DR2, -DR4, -DQ6, and -DQ8) (18). Furthermore, HIVBr18 promoted high magnitude, broad, and polyfunctional CD4^+^ and CD8^+^ T cell responses to 8 out of 18 vaccine-encoded peptides in BALB/c mice (19). This epitope-based vaccine concept may cope with HIV genetic variability, since it induces a broad T cell response focused on conserved HIV epitopes, and may also provide increased population coverage, given the promiscuity of HLA class II binding to multiple epitopes.

DNA vaccines are relatively easy and cheap to produce, being promising agents to control epidemics in remote, resource-poor locations (20). However, DNA vaccines have shown limited immunogenicity in non-human primates and in humans, possibly due to the low amount of the expressed antigen (21). For this reason, different approaches have been pursued in order to overcome this hurdle (22). Dendritic cells (DCs) have the ability to link innate and adaptive immunity because they are able to effectively acquire, process, and present a myriad of pathogen-derived epitopes mainly to T cells (23). In mouse spleen and lymph nodes, two major subtypes of resident DCs have been described: the CD11c^+^CD8α^+^ DCs that additionally express high levels of DEC205 endocytic receptor and the CD11c^+^CD8α^−^ that express the DCIR2 receptor (24, 25). *In vivo* antigen targeting to the CD11c^+^CD8α^+^ DCs was first demonstrated when two model antigens were fused to a monoclonal antibody (mAb) directed to the DEC205^+^ receptor. Ovalbumin and hen egg lysozyme were successfully coupled to the αDEC205 mAb, and effective presentation to either CD4^+^ or CD8^+^ T cells was observed, eliciting both robust humoral and cellular responses (26, 27). Different pathogen-derived antigens were shown to be efficiently processed and presented to T cells when targeted to the CD11c^+^CD8α^+^ DCs through αDEC205 mAb, such as *Plasmodium yoelii* (28), *Plasmodium falciparum* (29), *Trypanosoma cruzi* (30), *Mycobacterium tuberculosis* (31), HIV (32–34), and dengue virus (35). Furthermore, it was shown that targeting of HIV antigens using αDEC205 mAb could be an efficient vaccine platform. A single dose of αDEC205-Gag mAb in the presence of poly (I:C) induced protective CD4^+^ T responses when mice were challenged with recombinant vaccinia virus expressing Gag (33). In addition, αDEC205-p24 in the presence of poly (I:C) led to strong polyfunctional CD4^+^ profile that was able to induce proliferating and cytokine-producing T cells (32). HIV p24 targeted to CD11c^+^CD8α^+^ DCs also induced Th1 CD4^+^ T cells as well as cross-presentation to CD8^+^ T cells (36). Immunization with an anti-human DEC205-p24 mAb induced IFNγ- and IL-2-producing cells and was able to elicit high titers of anti-human IgG in transgenic mice (37). αDEC205-Gag targeting was also shown to assist a protective response to a DNA vaccine by mobilizing CD8^+^ T cells after challenge (38). More recently, αDEC205-p24 mAb was evaluated for intranasal immunization, and it was able to induce HIV-specific immunity in the gastrointestinal tract (34).

In recent years, evidence has shown that heterologous prime-boost vaccination was an effective strategy to generate powerful antibody responses (39, 40), to improve the magnitude and quality of T cell responses (41), and to induce protection against different pathogens (42), including HIV. We thus hypothesized that targeting HIV CD4^+^ T cell epitopes to DCs using the αDEC205 mAb would be able to induce higher specific cellular responses against HIV-1 when compared to a DNA vaccine encoding the same epitopes. In the current study, we assessed the polyfunctionality of HIV-specific T cell responses induced by αDECHIVBr8 chimeric mAb and the DNA vaccine HIVBr8 in homologous and heterologous prime-boost immunization regimens. Our results showed that immunization with αDECHIVBr8 solely or heterologous prime-boost with HIVBr8 followed by αDECHIVBr8 was able to induce broader and polyfunctional CD4^+^ and CD8^+^ T cells when compared to the DNA vaccine alone.

## Materials and Methods

### Epitopes

The sequences of HIV-1 epitopes selected for this study were previously described by Fonseca et al. (16) and are the following: p6 (32–46), p17 (73–89), pol (785–799), gp160 (188–201), rev (11–27), vpr (65–82), vif (144–158), and nef (180–194) (Table 1). These epitopes were derived from the previously described DNA vaccine HIVBr18 (18, 19) and comprise the eight mentioned epitopes (HIVBr8) that can bind to I-Ad and are recognized by T cells from immunized BALB/c mice. The epitopes were assembled *in tandem* and are separated by GPGPG at C and N termini to avoid the creation of junctional epitopes that may interfere with processing and presentation (43).

**Table 1:** **Amino acid sequence of HIV epitopes**.

Epitope	Sequence
p6 (32–46)	DKELYPLASLRSLFG
p17 (73–89)	EELRSLYNTVATLYCVH
pol (785–799)	GKIILVAVHVASGYI
gp160 (188–201)	NTSYRLISCNTSVI
rev (11–27)	ELLKTVRLIKFLYQSNP
vpr3 (65–82)	QQLLFIHFRIGCRHSRIG
vif (144–158)	SLQYLALVALVAPKK
nef (180–194)	VLEWRFDSRLAFHHV

*The sequences of HIV-1 epitopes selected for this study were previously described by Fonseca et al. (16). These epitopes were derived from the previously described DNA vaccine HIVBr18 (18, 19) and comprise the eight mentioned epitopes (HIVBr8) that can bind to I-A^d^ and are recognized by T cells from immunized BALB/c mice*.

### Cloning the DNA Sequence Encoding HIV-1 Epitopes: pVAXHIVBr8 Generation

The HIVBr8 nucleotide sequence was codon optimized, and a Kozak sequence was included at the 5′ end to improve mammalian expression. The artificial gene (Genscript) was cloned between the *Hin*dIII and *Xho*I restriction sites of the pVAX1 vector (Invitrogen) to generate the pVAXHIVBr8 plasmid that was amplified using DH5α cells. The pVAXHIVBr8 was purified using the Endofree Plasmid Giga Kit (Qiagen) according to the manufacturer’s instructions. The yield and quality of purified DNA was determined by spectrophotometry at 260 nm and confirmed by agarose gel electrophoresis.

### Fusion of HIV-Derived CD4^+^ T Cell Epitopes to the αDEC205 Antibody: αDECHIVBr8 mAb Generation

Plasmids encoding the light and heavy chains of the mouse αDEC205 antibody were kindly provided by Dr. Michel C. Nussenzweig (The Rockefeller University). The artificial HIVBr8 gene was produced (Genscript), digested from pUC57 vector, and cloned in frame with the carboxyl terminus of the heavy chain of the mouse DEC205 (clone NLDC145) between the 5′ *Xho*I and 3′ *Not*I sites. Large-scale preparation of plasmids pDECHIVBr8, empty pDEC (a negative non-fused control), and pDEC kappa (encoding the αDEC205 kappa light chain) were prepared using Maxi Plasmid Purification Kit (Qiagen), according to the manufacturer’s instructions. The yield and quality of purified DNA were determined by spectrophotometry at 260 nm and confirmed by agarose gel electrophoresis.

### Expression and Purification of αDECHIVBr8 mAb

The chimeric αDEC and αDECHIVBr8 mAbs were produced and purified after transfection of human embryonic kidney 293T cells (ATCC, CRL-11268) exactly as described (35).

### Immunoblot

Approximately 1 µg of the αDEC and αDECHIVBr8 mAbs were run on 12% SDS-PAGE gels under reducing conditions, and subsequently transferred to nitrocellulose membranes (GE Healthcare) at 100 V for 90 min in transfer buffer (glycine 39 mM, Tris 48 mM, SDS 10% and methanol 20%, pH 8.3). After transfer, nitrocellulose membranes were blocked in PBS 0.02% Tween-20 (PBST), 5% non-fat milk, and 2.5% BSA, overnight at 4°C. Membranes containing the reduced mAbs were then incubated with peroxidase-labeled goat anti-mouse IgG Fc specific (1:5,000, Jackson Laboratories) plus peroxidase-labeled goat anti-mouse IgG kappa (1:3,000, SouthernBiotech) for 60 min at room temperature. After three washes with PBST, the reaction was developed using chemiluminescence (ECL kit, GE Healthcare) and captured on Kodak film.

### Binding Assay

Binding assays were performed using CHO cells expressing either the mouse DEC205 (CHOmDEC) or DCIR2 (CHOmDCIR2) receptors, kindly provided by Dr. Michel Nussenzweig (The Rockefeller University). Purified mAbs were diluted to 4, 2, or 1 µg/mL and incubated with the CHOmDEC or CHOmDCIR2 cells at 4°C for 30 min, exactly as described by Henriques et al. (35). Next, the cells were washed and incubated with anti-mouse IgG1-PE (clone A85-1, BD Biosciences) for 30 min at 4°C. Additionally, 4, 2, or 1 µg/mL of αDEC or αDECHIVBr8 mAbs were incubated with 5 million splenocytes at 4°C for 40 min and then incubated with anti-CD49b-biotin (clone DX5), anti-CD19-biotin (clone 1D3), anti-CD3-biotin (clone 145-2C11), Streptavidin-APCCy7, anti-CD11c-APC (HL3), anti-IAIE-FITC (clone 2G9), anti-CD8-Pacific Blue (clone 53-6.7), and anti-IgG1-PE (clone A85-1). All monoclonal antibodies were purchased from BD Biosciences. Fifty thousand events were acquired for the analysis of binding to CHO cells and 3 million for the analysis of binding to splenocytes. Samples were acquired using FACS Canto II flow cytometer (BD Biosciences) and analyzed using the FlowJo software (version 9.9, Tree Star, San Carlos, CA, USA).

### Animals and Immunization

The 6- to 8-week-old female BALB/c (H-2^d^) mice were purchased from Centro de Desenvolvimento de Modelos Experimentais para Medicina e Biologia (CEDEME), Brazil. Groups of six animals were immunized with two doses—2 weeks apart—of 4 µg of αDECHIVBr8 mAb in the presence of 50 µg of adjuvant poly (I:C) (Invivogen) delivered intraperitoneally (IP) or subcutaneously (SC), or with two doses of 100 µg of the DNA vaccine pVAXHIVBr8 by intramuscular route (IM). The control groups were immunized with 4 µg of αDEC in the presence of 50 µg of poly (I:C) or with pVAX (empty vector). Furthermore, for heterologous prime-boost regimen, other groups received one dose of the mAb followed by one dose of DNA vaccine or *vice versa*. The control groups were immunized with one dose of αDEC mAb together with poly (I:C) followed by one dose with pVAX or *vice versa*.

### Spleen Cell Isolation for Immune Assays

Two weeks after the last immunization, mice were euthanized and spleens were removed aseptically. After obtaining single cell suspensions, cells were washed in 10 mL of RPMI 1640 (Gibco). Cells were then resuspended in R-10 [RPMI supplemented with 10% of fetal bovine serum (Gibco)], 2 mM L glutamine (Gibco), 10 mM Hepes (Gibco), 1 mM sodium pyruvate (Gibco), 1% v/v non-essential amino acids solution (Gibco), 40 µg/mL of gentamicin, 20 µg/mL of peflacin, and 5 × 10^−5^ M 2-mercaptoethanol (Gibco). The viability of cells was evaluated using 0.2% Trypan Blue exclusion dye to discriminate between live and dead cells. Cell concentration was estimated with the aid of a cell counter (Countess, Invitrogen) and adjusted in cell culture medium.

### T Cell ELISpot Assay

Splenocytes from immunized mice were obtained as previously described and assayed for their ability to secrete IFNγ after *in vitro* stimulation with 5 µM of individual or pooled HIV-1 peptides using the ELISpot assay. The ELISpot assay was performed using mouse IFNγ ELISpot Ready-SET-Go! (eBiosciences) according to the manufacturer’s instructions. Spots were counted using an AID ELISpot Reader System (Autoimmun Diagnostika GmbH, Germany). The cutoff was 15 SFU per million splenocytes.

### Analysis of Polyfunctional HIV-Specific T Cell Responses by Multiparametric Flow Cytometry

To analyze HIV-specific T cell expansion, proliferation, and cytokine production, splenocytes from immunized mice were labeled with carboxyfluorescein succinimidyl ester (CFSE) (19). In summary, freshly isolated splenocytes were resuspended (50 × 10^6^/mL) in PBS and labeled with 1.25 µM of CFSE (Molecular Probes) at 37°C for 10 min. The reaction was quenched with RPMI 1640 supplemented with 10% FBS (R10), and cells were washed with R10 before resuspension in RPMI 1640. Cells were cultured in 96-well round-bottomed plates (5 × 10^5^/well in triplicates) for 5 days at 37°C and 5% CO_2_ with medium only or pooled HIV-1 peptides (5 µM). After 4 days of incubation, cells were restimulated in the presence of 2 µg/mL anti-CD28 (BD Pharmingen), 5 µM of individual or pooled HIV-1 peptides and brefeldin A GolgiPlug™ (BD Pharmingen) for further 12 h. After the incubation period, cells were washed with FACS buffer (PBS with 0.5% BSA and 2 mM EDTA) and surface stained with anti-CD3 APCCy7 (clone 145-2C11), anti-CD4 PerCP (clone RM4-5), and anti-CD8 Pacific Blue (clone 53-6.7) monoclonal antibodies for 30 min at 4°C. Cells were fixed and permeabilized using Cytofix/Cytoperm™ kit (BD Pharmingen), according to the manufacturer’s instructions. After permeabilization, cells were washed with Perm/Wash buffer (BD Biosciences) and stained intracellularly with anti-IL2 PE (clone JES6-5H4), anti-TNFα PECy7 (clone MP6-XT22), and anti-IFNγ APC (clone XMG1.2) monoclonal antibodies for 30 min at 4°C. Following staining, cells were washed twice and resuspended in FACS buffer. All antibodies were from BD Pharmingen. Samples were acquired on a FACSCanto II flow cytometer (BD Biosciences) and then analyzed using FlowJo software (version 9.9, Tree Star, San Carlos, CA, USA). To analyze cellular polyfunctionality, we used the Boolean gating platform (FlowJo software) to create several combinations of the three cytokines (IL-2, TNFα, and IFNγ) within the CFSE^low^ population resulting in seven distinct patterns. The percentages of cytokine-producing cells were calculated by subtracting background values. For each experiment performed, unstained and all single-color controls were processed to allow proper compensation.

### Data Analysis

Statistical significance (*p*-values) was calculated by using a two-way ANOVA and Bonferroni’s or one-way ANOVA and Tukey honest significant difference. Statistical analysis and graphical representation of data was performed using GraphPad Prism version 5.0 software.

### Ethics Statement

Mice were housed and manipulated under SPF conditions in the animal care facilities of the Division of Immunology, Federal University of São Paulo (UNIFESP). This study was carried out in accordance with the recommendations of the National Institutes of Health Guide for the Care and Use of Laboratory Animals and the Brazilian National Law (11.794/2008). The protocol (number 3226180814) was approved by the Institutional Animal Care and Use Committee (CEUA) of Federal University of São Paulo.

## Results

### αDECHIVBr8 mAb Binds Specifically the DEC205 Receptor

**Figure 1:**
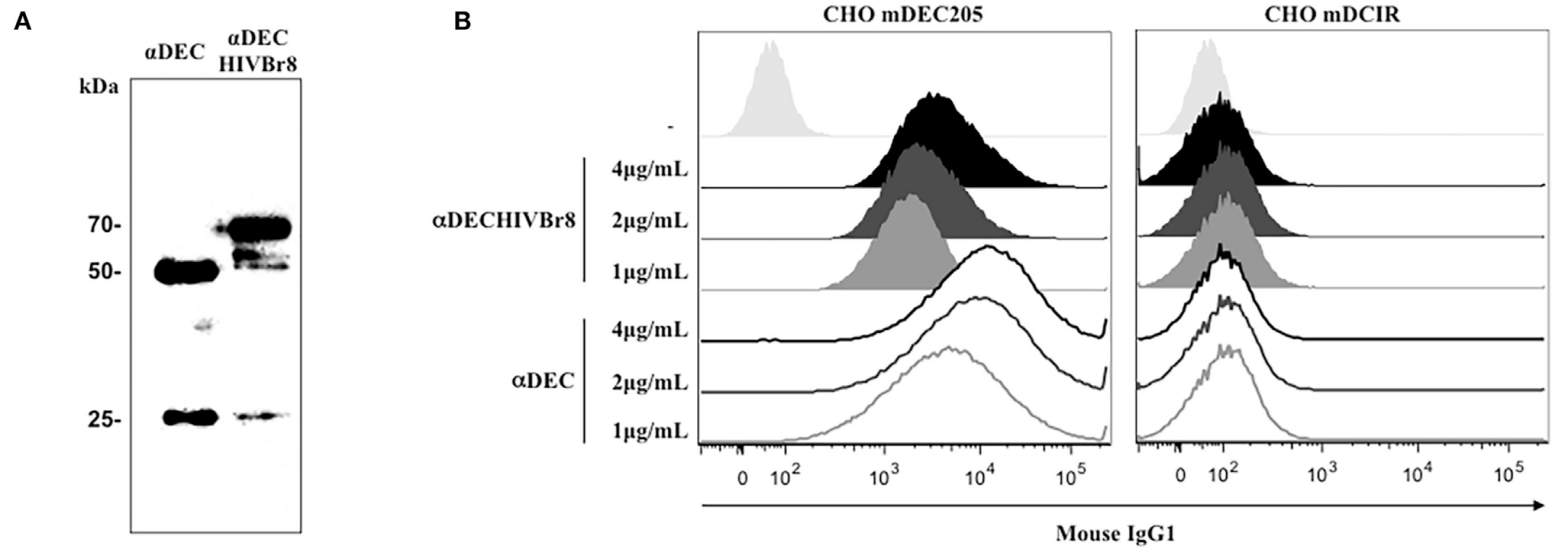

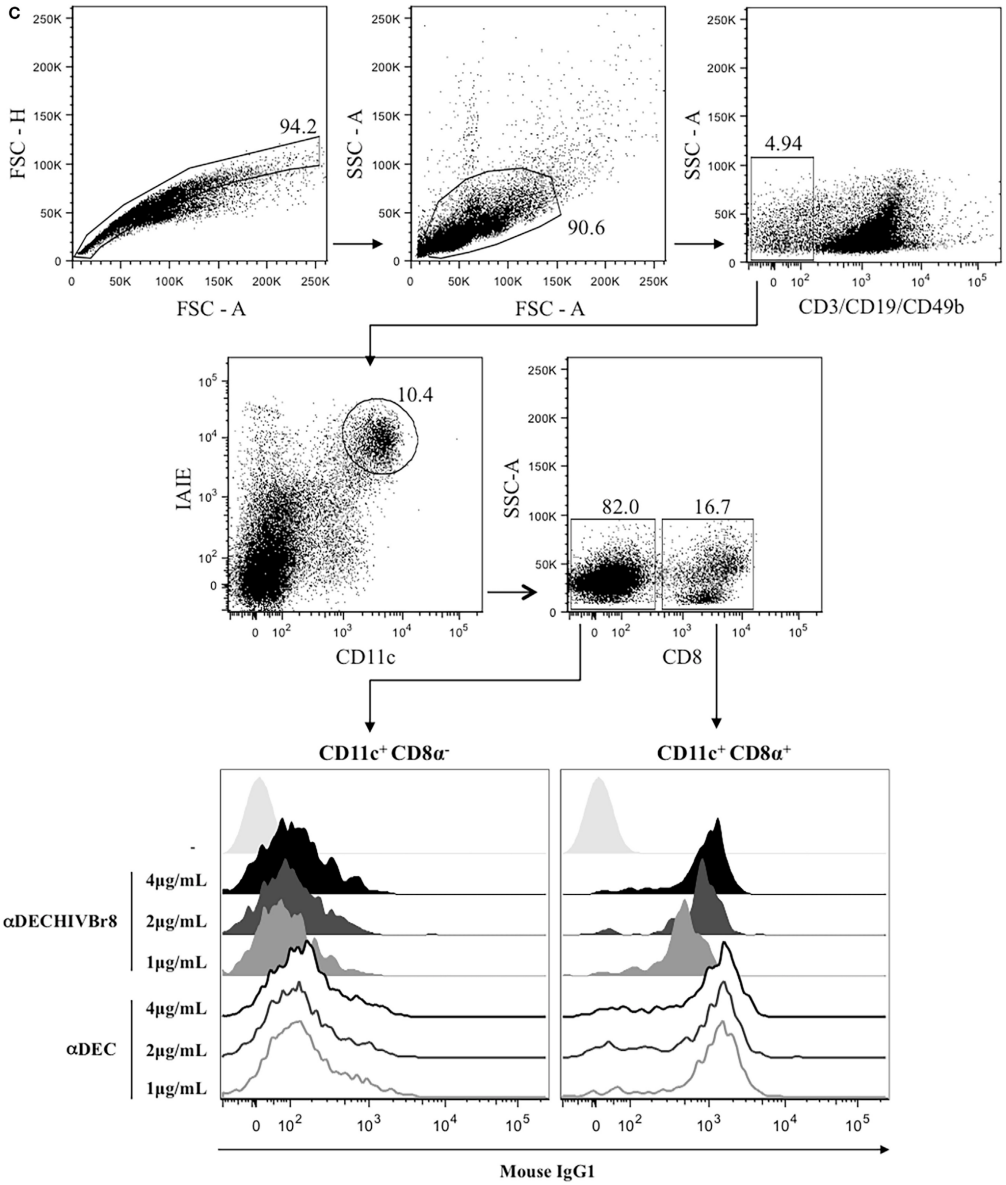
**The chimeric αDECHIVBr8 was successfully produced and retained its ability to bind to cells expressing the DEC205 receptors**. **(A)** One microgram of each monoclonal antibody (mAb) was run on 12% SDS-PAGE under reducing conditions. An immunoblot was performed using peroxidase-labeled goat anti-mouse IgG Fc specific and peroxidase-labeled goat anti-mouse IgG kappa. Molecular weight (kilodaltons), αDEC (control), and αDECHIVBr8; **(B)** CHO cells expressing either DEC205 (left) or DCIR2 (right) receptors were incubated with 4, 2, or 1 µg/mL of αDEC (control) or αDECHIVBr8, following staining with anti-mouse IgG1 PE antibody. Fifty thousand events were acquired in FACS Canto II and analysis was performed using FlowJo software; **(C)** 5 million splenocytes from BALB/c mice were incubated with 4, 2, or 1 µg/mL of the chimeric αDECHIVBr8 or αDEC mAbs. Splenocytes were then incubated with a pool of fluorescent antibodies and gated as singlets and CD3^−^CD19^−^CD49b^−^. Dendritic cells were selected as CD11c^+^ IAIE^+^ and subsequently divided into CD8α^+^ and CD8α^−^. Binding was detected on 3 × 10^6^ cells using an anti-mouse IgG1-PE antibody. Analysis was performed using FlowJo software.

In an attempt to induce a T cell response against HIV, we cloned eight CD4^+^ T cell epitopes in fusion with the heavy chain of the DEC205 receptor. αDECHIVBr8 and control αDEC205 mAbs were purified and analyzed in 12% SDS polyacrylamide gel under reducing conditions. Figure 1A shows an immunoblot in which both mAbs were transferred to a nitrocellulose membrane and incubated with anti-mouse total IgG and anti-mouse IgG kappa chain. Two bands that correspond to the heavy chain (~50 kDa) and to the light chain were detected. In the αDECHIVBr8 mAb preparation, we detected the light chain (~25 kDa) and also a band of ~70 kDa that corresponds to the heavy chain of αDEC205 fused with the HIVBr8 sequence. Next, we tested whether the αDECHIVBr8 mAb retained its binding capacity to either CHO cells expressing the mouse DEC205 receptor or to the CD11c^+^CD8α^+^ spleen DCs. Figure 1B shows that αDECHIVBr8 mAb was able to specifically bind to CHO cells expressing the mouse DEC205 receptor in a dose-dependent manner but not to CHO cells expressing the mouse DCIR2 receptor. The control αDEC205 mAb showed the same binding pattern. More interestingly, the αDECHIVBr8 mAb was able bind specifically to the murine CD11c^+^CD8α^+^ DCs but not to the CD11c^+^CD8α^−^ DCs, showing its specificity for DCs that express DEC205 *in vivo*. As expected, the control αDEC205 mAb also bound to the CD11c^+^CD8α^+^ DCs (Figure 1C). Taken together, these results showed that the αDECHIVBr8 mAb was successfully produced and retained its capacity to bind to murine DCs expressing DEC205.

### Immunization with the αDECHIVBr8 mAb Induces Higher Immune Responses than the pVAXHIVBr8 DNA Vaccine

**Figure 2:**
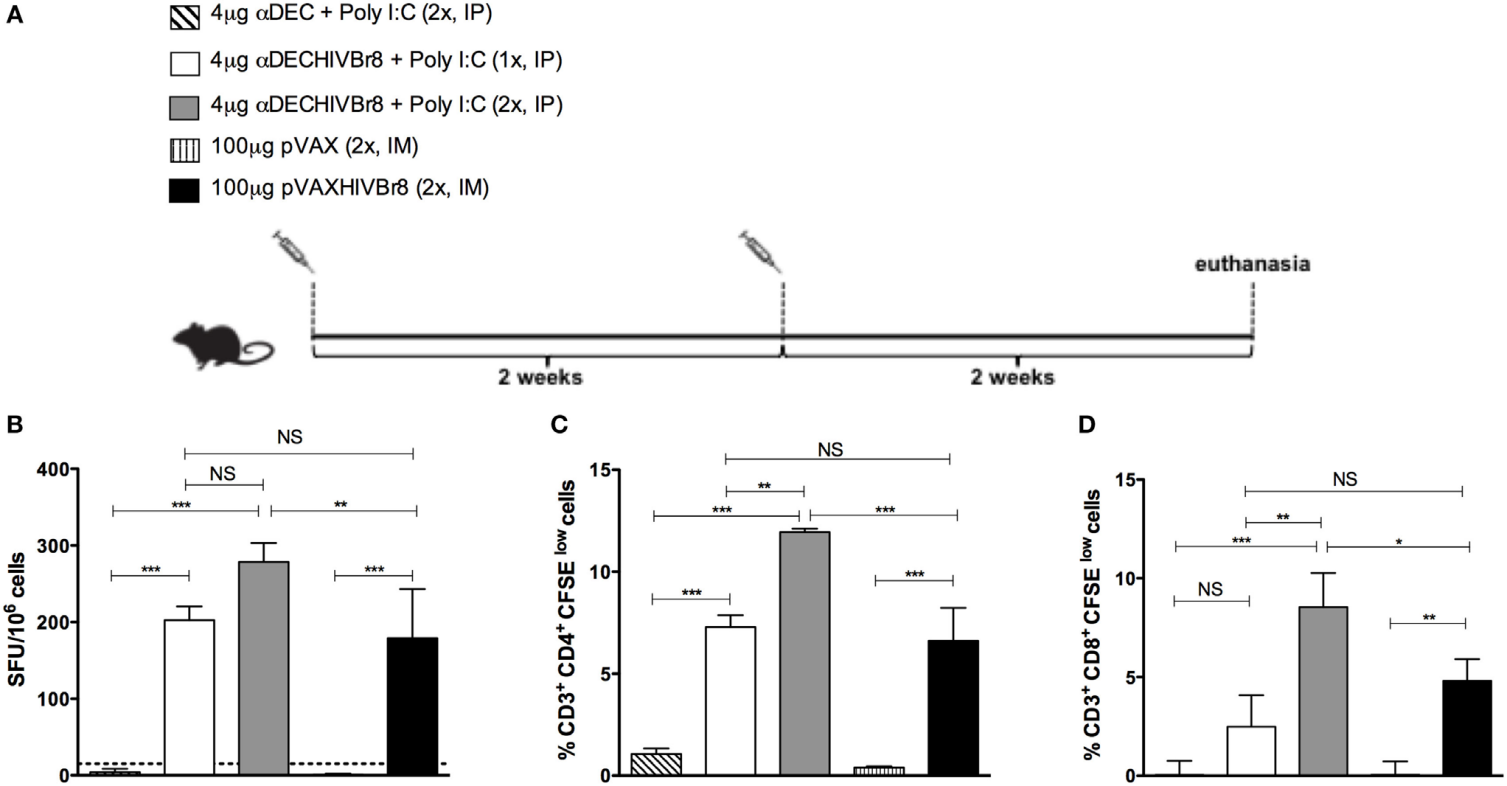
**Immunization with the chimeric αDECHIVBr8 monoclonal antibody induces higher immune responses when compared to immunization with the pVAXHIVBr8 DNA vaccine**. BALB/c mice (*n* = 6) were immunized with one or two doses of 4 µg of αDEC or αDECHIVBr8 in the presence of poly (I:C) adjuvant (IP) or two doses of 100 µg of pVAX or pVAXHIVBr8 DNA vaccine (IM). **(A)** Immunization scheme. Fifteen days after the second dose, the spleen of each animal was removed and the splenocytes **(B)** were cultured in the presence of pooled HIV-1 peptides (5 µM) for 18 h to evaluate the number of IFN-γ-producing cells by ELISpot assay. SFU, spot forming units. Cutoff = 15 SFU/10^6^ cells and is represented by the dotted line. **(C,D)** Splenocytes were labeled with carboxyfluorescein succinimidyl ester (CFSE) (1.25 µM) and cultured in the presence of pooled HIV-1 peptides (5 µM) for 5 days to evaluate specific proliferation. After staining with fluorochrome-labeled anti-CD3, anti-CD4, and anti-CD8 monoclonal antibodies, cells were analyzed by flow cytometry. CFSE dilution on gated CD3^+^CD4^+^ **(C)** or CD3^+^CD8^+^ **(D)** cells was used as readout for antigen-specific proliferation. One million events were acquired in a live lymphocyte gate. The percent of proliferating CD4^+^ and CD8^+^ CFSE^low^ cells was determined in the CD3^+^ cell population. The percentage of proliferating T cells was calculated subtracting by the values of stimulated from non-stimulated cultures. NS, not significant; *p < 0.05; **p < 0.01; ***p < 0.001. Data represent mean ± SD.

We initially evaluated the cellular immune responses against the pooled HIV peptides in BALB/c mice immunized with one or two doses of αDECHIVBr8 mAb (4 µg) in the presence of poly (I:C) and compared to two doses of the pVAXHIVBr8 DNA vaccine (100 µg) (Figure 2A). Splenocytes from mice immunized with two doses of the αDECHIVBr8 mAb presented a higher number of specific IFNγ-producing cells when compared to mice immunized with two doses of the DNA vaccine (about 280 and 180 SFU/10^6^, respectively). In addition, no significant difference was observed when the groups that received one or two doses of the αDECHIVBr8 mAb were compared (Figure 2B). Notably, when specific cellular proliferation was evaluated, mice that received two doses of αDECHIVBr8 mAb displayed higher frequency of specific CD4^+^ (12%, Figure 2C) and CD8^+^ (8.5%, Figure 2D) T cells that proliferated when compared to all the other groups. Significantly, these were almost twofold higher than the numbers found in the group receiving two doses of pVAXHIVBr8 (6.6% CD4^+^ and 4.8% specific CD8^+^ T cells). Control groups that were immunized with αDEC mAb or pVAX plasmid did not show specific IFNγ production or T cell proliferation. Of note, the number of CD4^+^ and CD8^+^ T cells that proliferated in mice immunized with two doses of the αDECHIVBr8 mAb was higher than the number detected in mice immunized with just one dose. These results led us to conclude that two doses are more effective to induce higher immune responses.

**Figure 3:**
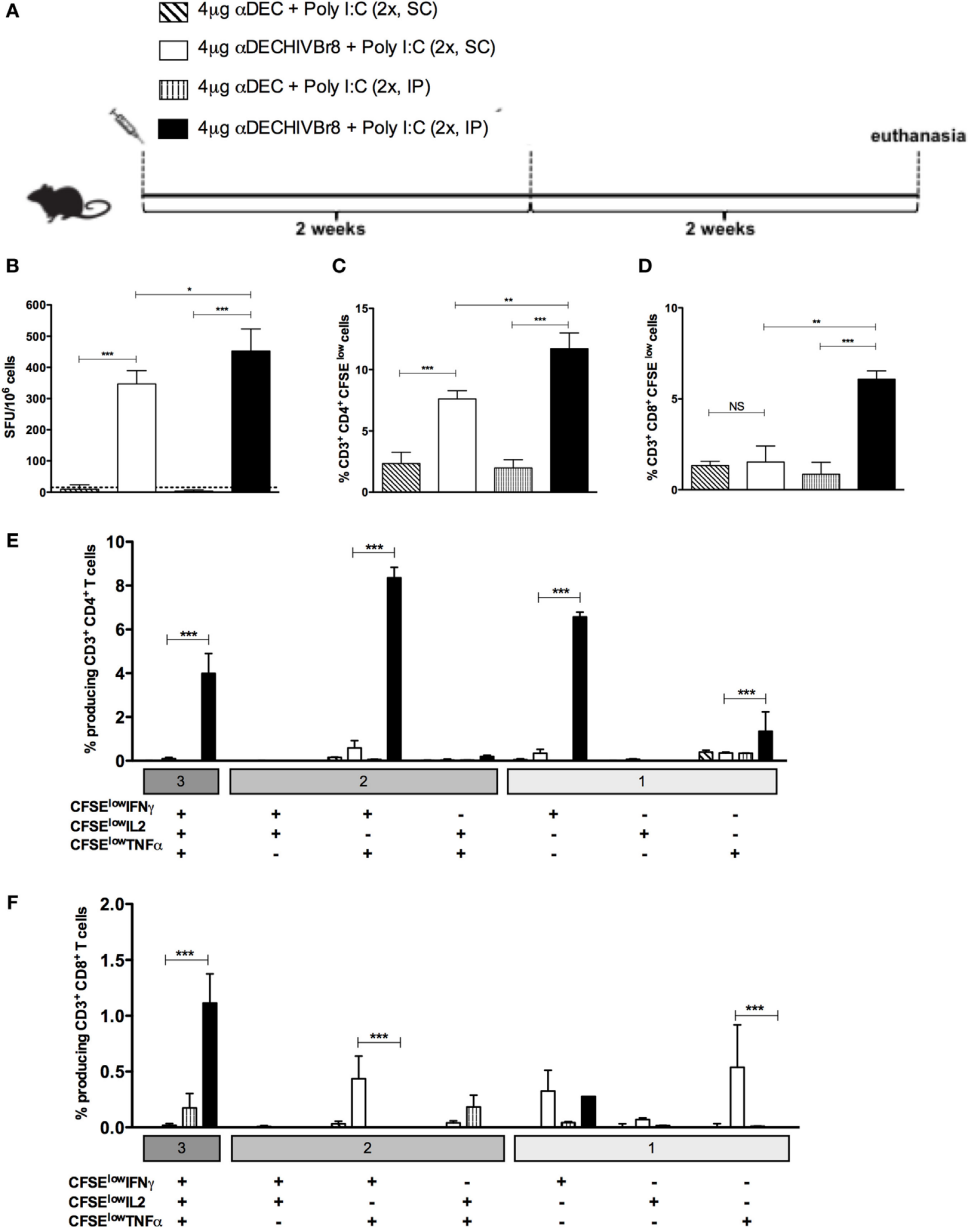
**The intraperitoneal route elicits higher T cell responses in the spleen than subcutaneous administration of the chimeric αDECHIVBr8 monoclonal antibody**. BALB/c mice (*n* = 6) were immunized with two doses of 4 µg of αDEC or αDECHIVBr8 in the presence of poly (I:C) adjuvant by intraperitoneal (IP) or subcutaneous (SC) routes. **(A)** Immunization scheme. Fifteen days after the second dose, the spleen of each animal was removed and the splenocytes **(B)** were cultured in the presence pooled HIV-1 peptides (5 µM) for 18 h to evaluate the number of IFN-γ-producing cells by ELISpot assay. SFU, spot forming units. Cutoff = 15 SFU/10^6^ cells and is represented by the dotted line. **(C,D)** Splenocytes were labeled with carboxyfluorescein succinimidyl ester (CFSE) (1.25 µM) and cultured in the presence of pooled HIV-1 peptides (5 µM) for 5 days to evaluate specific proliferation. After staining with fluorochrome-labeled anti-CD3, anti-CD4, and anti-CD8 monoclonal antibodies, cells were analyzed by flow cytometry. CFSE dilution on gated CD3^+^CD4^+^ **(C)** or CD3^+^CD8^+^ **(D)** cells was used as readout for antigen-specific proliferation. For detection of cytokine-producing T cells, cells were pulsed on day 4 for 12 h with pooled peptides in the presence of anti-CD28 and brefeldin A. Cells were then surface stained with anti-CD3, CD4, and CD8, permeabilized, and stained for intracellular cytokines. Multiparameter flow cytometry was used to determine the frequency of IFNγ-, IL2-, or TNFα-producing CD4^+^ and CD8^+^ T cells **(E,F)**. After gating on proliferating (CFSE^low^) and cytokine-producing cells, Boolean combinations were then created using FlowJo software to determine the frequency of each response based on all possible combinations of cytokine-producing CD4^+^ **(E)** and CD8^+^ **(F)** T cells. One million events were acquired in a live lymphocyte gate. The percentage of proliferating and cytokine-producing T cells was calculated subtracting the values of stimulated from non-stimulated cultures. NS, not significant; *p < 0.05; **p < 0.01; ***p < 0.001. Data represent mean ± SD.

Next, we decided to address if the route of immunization would alter the efficacy of the αDECHIVBr8 mAb immunization. For that purpose, mice were immunized with two doses of the αDEC or αDECHIVBr8 in the presence of poly (I:C) by intraperitoneal (IP) or subcutaneous (SC) route (Figure 3A). As shown in Figure 3B, IP immunization with the αDECHIVBr8 mAb was able to induce higher IFNγ-producing cells than the SC immunization. Similar results were obtained when we measured the percentage of specific proliferating CD4^+^ and CD8^+^ T cells (Figures 3C,D, respectively). Control mice immunized with αDEC205 mAb did not show significant production of IFNγ or proliferation independently of the route used. We subsequently characterized the profile of polyfunctional T cells. Using multiparameter flow cytometry, we detected antigen-specific T cells (CD4^+^ and CD8^+^) based on their ability to proliferate (CFSE dilution assay) and produce the effector cytokines IFNγ, TNFα, and IL2 simultaneously. Boolean combinations of proliferating and cytokine-positive populations indicated that immunization by IP route was most effective to induce higher percentage CD4^+^ T cells that proliferated and produced simultaneously IFNγ/IL2/TNFα or IFNγ/TNFα or TNFα only (Figure 3E). Also, IP immunization induced a higher percentage of CD8^+^ T cells that proliferated and produced IFNγ/IL2/TNFα simultaneously when compared to the group immunized by SC route (Figure 3F). We can conclude that immunization using two doses of the αDECHIVBr8 mAb by the IP route was better to induce T cell responses with greater magnitude and more polyfunctional than those induced by the pVAXHIVBr8 DNA vaccine encoding the same epitopes.

### Homologous or Heterologous αDECHIVBr8 Prime-Boost Enhances IFN-γ Production, T Cell Proliferation, and Polyfunctional T Cells

**Figure 4:**
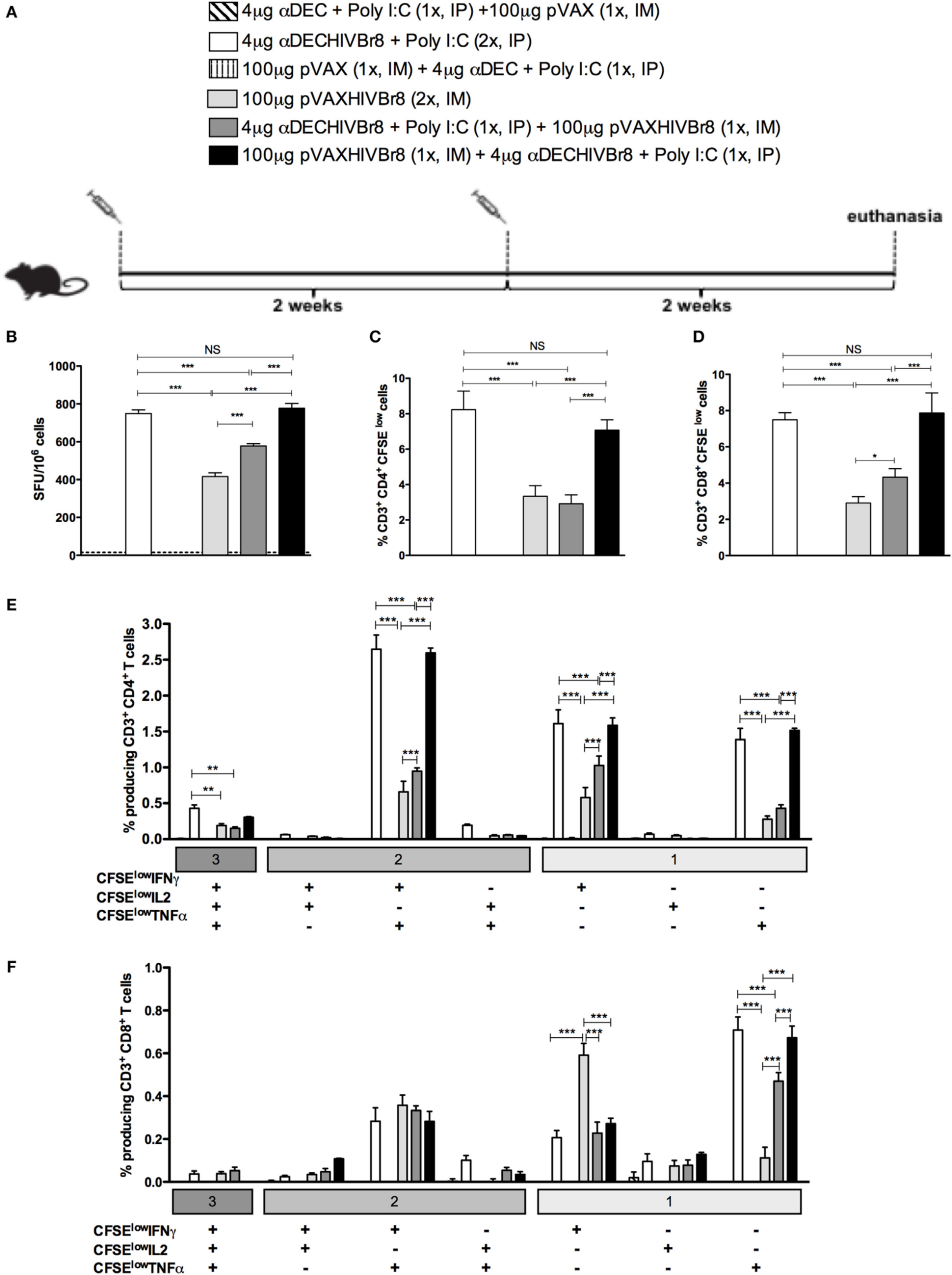
**Homologous αDECHIVBr8 monoclonal antibody (mAb) immunization or heterologous prime-boost induces polyfunctional T cell responses**. BALB/c mice (*n* = 6) were immunized with two doses of 4 µg of αDECHIVBr8 in the presence of poly (I:C) adjuvant (IP) or two doses of 100 µg of pVAXHIVBr8 DNA vaccine (IM). For heterologous regimens, mice were immunized with one dose of αDECHIVBr8 followed by one dose of DNA vaccine or *vice versa*. The control groups were immunized with one dose of αDEC mAb together with poly (I:C) followed by one dose with pVAX or *vice versa*. **(A)** Immunization scheme. Fifteen days after the second dose, the spleen of each animal was removed and the splenocytes **(B)** were cultured in the presence of pooled HIV-1 peptides (5 µM) for 18 h to evaluate the number of IFN-γ-producing cells by ELISpot assay. SFU, spot forming units. Cutoff = 15 SFU/10^6^ cells and is represented by the dotted line. **(C,D)** Splenocytes were labeled with carboxyfluorescein succinimidyl ester (CFSE) (1.25 µM) and cultured in the presence of pooled HIV-1 peptides (5 µM) for 5 days to evaluated specific proliferation. After staining with fluorochrome-labeled anti-CD3, anti-CD4, and anti-CD8 monoclonal antibodies, cells were analyzed by flow cytometry. CFSE dilution on gated CD3^+^CD4^+^ **(C)** or CD3^+^CD8^+^ **(D)** cells was used as readout for antigen-specific proliferation; For detection of cytokine-producing T cells, cells were pulsed on day 4 for 12 h with pooled peptides in the presence of anti-CD28 and brefeldin A. Cells were then surface stained with anti-CD3, CD4, and CD8, permeabilized, and stained for intracellular cytokines. Multiparameter flow cytometry was used to determine the frequency of IFNγ-, IL2-, or TNFα-producing CD4^+^ and CD8^+^ T cells **(E,F)**. After gating on proliferating (CFSE^low^) and cytokine-producing cells, Boolean combinations were then created using FlowJo software to determine the frequency of each response based on all possible combinations of cytokine-producing CD4^+^ **(E)** and CD8^+^ **(F)** T cells. One million events were acquired in a live lymphocyte gate. The percentage of proliferating and cytokine-producing T cells was calculated subtracting the values of stimulated from non-stimulated cultures. NS, not significant; *p < 0.05; **p < 0.01; ***p < 0.001. Data represent mean ± SD.

To test whether a heterologous prime-boost strategy would work with chimeric mAb, groups of mice were then immunized with one dose of αDECHIVBr8 mAb (prime) followed by one dose of the pVAXHIVBr8 DNA vaccine (boost), or *vice versa*, and compared to the homologous prime-boost immunization strategy (two doses of chimeric mAb or DNA vaccine) (Figure 4A). Two weeks after the boost, splenocytes from immunized mice were incubated with pooled peptides and specific IFNγ production was measured by ELISpot assay. We detected the highest numbers of IFNγ-producing cells against the pooled peptides in mice that received two doses of αDECHIVBr8 and in the group receiving pVAXHIVBr8 priming followed by αDECHIVBr8 mAb boosting (Figure 4B). Interestingly, mice primed with αDECHIVBr8 mAb and boosted with pVAXHIVBr8 showed a lower response when compared to the two previously described groups. Finally, mice immunized with two doses of pVAXHIVBr8 presented the lowest number of IFNγ-producing cells (416 SFU/10^6^ cells, Figure 4B). A similar pattern was observed when we analyzed the percent of CD4^+^ and CD8^+^ specific proliferation (Figures 4C,D, respectively): mice immunized with two doses of αDECHIVBr8 mAb displayed 8.23% of CD4^+^ and 7.49% of CD8^+^ T cell specific proliferation, while pVAXHIVBr8 (prime)/αDECHIVBr8 (boost) displayed 7.04 and 7.86%, respectively. Lower percentages were observed in mice immunized with two doses of pVAXHIVBr8 or αDECHIVBr8 mAb (prime)/pVAXHIVBr8 (boost). In addition, we characterized the phenotype and functionality of antigen-specific CD4^+^ and CD8^+^ T cells based on their ability to proliferate (CFSE^low^) and produce IFNγ, TNFα, and IL2 individually or in combinations (Figure S1 in Supplementary Material). Figure 4E shows once more that immunization with two doses of αDECHIVBr8 and pVAXHIVBr8 (prime)/αDEC205-HIVBr8 (boost) were most effective to induce higher percentage of CD4^+^ T cells that proliferated and produced simultaneously IFNγ/TNFα or IFNγ only or TNFα only. When we analyzed the CD8^+^ T cells, two doses of pVAXHIVBr8 DNA vaccine showed a higher percentage of CD8^+^ T cell proliferating and producing only IFNγ when compared to the other groups (Figure 4F). By contrast, CD8^+^ T cells from mice immunized with two doses of αDECHIVBr8 and pVAXHIVBr8 (prime)/αDECHIVBr8 (boost) were able to proliferate and mainly produce TNFα. αDEC and pVAX immunized mice displayed negligible percentages of specific proliferating/cytokine-producing T cells. Extended comparative analysis revealed that αDECHIVBr8 and αDECHIVBr8 (prime)/pVAXHIVBr8 (boost) immunized mice displayed higher frequency of non-proliferating (CFSE^high^) IFNγ^+^/IL2^+^/TNFα^+^ and CFSE^high^ IFNγ^+^/TNFα^+^-producing CD4^+^ T cells when compared to other groups (Figure S2A in Supplementary Material). Analysis of CD8^+^ T cell compartment demonstrated that αDECHIVBr8 (prime)/pVAXHIVBr8 (boost) was the most efficient strategy to induce CFSE^high^ IFNγ^+^/IL2^+^/TNFα^+^ and CFSE^high^ IFNγ^+^ cells, while αDECHIVBr8 immunization induced higher frequency of CFSE^high^ TNFα^+^ cells (Figure S2B in Supplementary Material). Taken together, these results showed that homologous immunization with αDECHIVBr8 mAb or heterologous pVAXHIVBr8 (prime)/αDECHIVBr8 (boost) were able to induce broad specific cellular responses, and polyfunctional CD4^+^ and CD8^+^ T cells that proliferated and produced effector Th1 cytokines to epitopes encoded by the chimeric mAb and the DNA vaccine.

**Figure 5:**
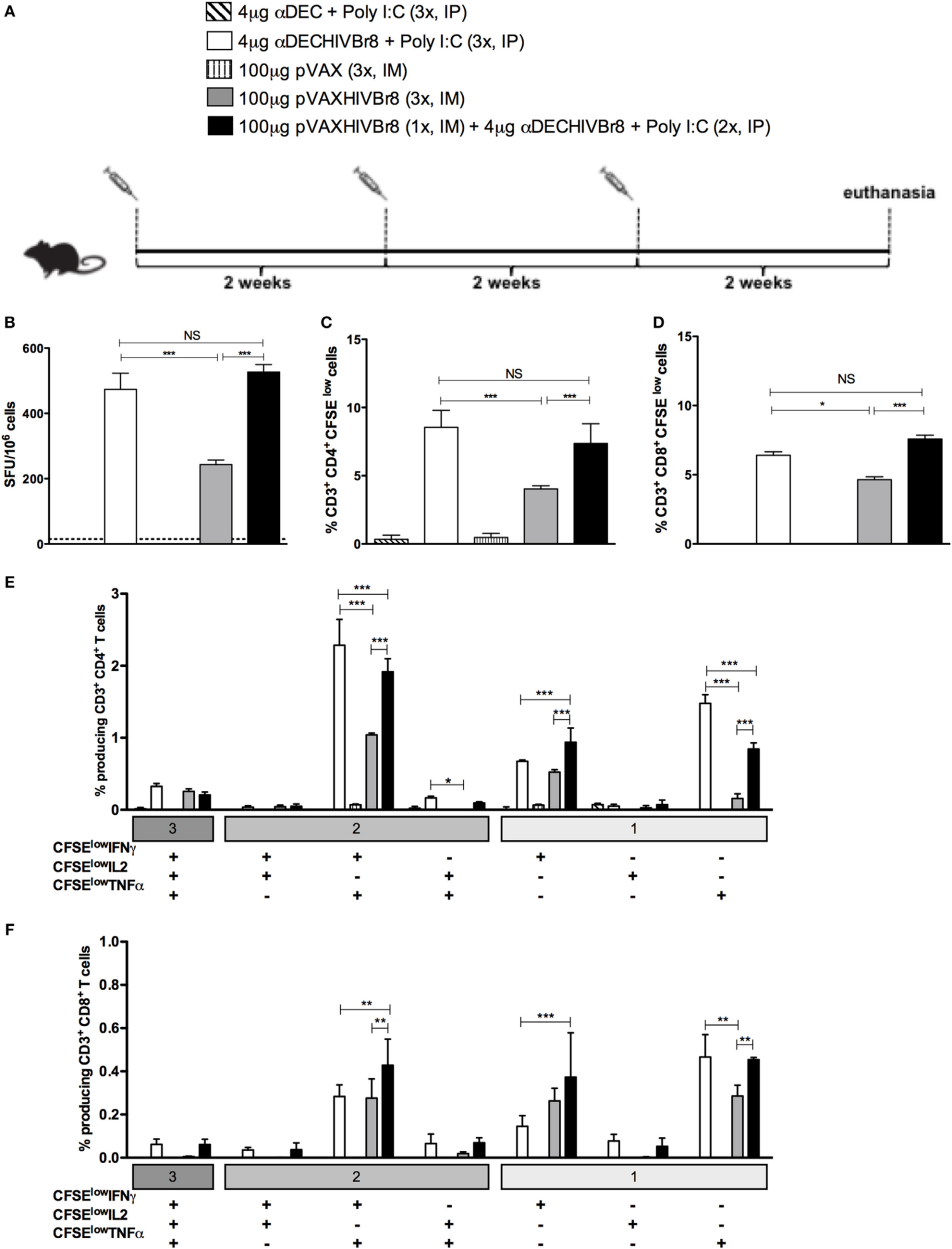
**The chimeric αDECHIVBr8 monoclonal antibody (mAb) is more immunogenic even with increased doses of the DNA vaccine**. BALB/c mice (*n* = 6) were immunized with three doses of 4 µg of αDECHIVBr8 in the presence of poly (I:C) adjuvant (IP) or three doses of 100 µg of pVAXHIVBr8 DNA vaccine (IM). For heterologous regimens, mice were immunized with one dose of DNA vaccine followed by two doses of the chimeric mAb. **(A)** Immunization scheme. Fifteen days after the second dose, the spleen of each animal was removed and the splenocytes **(B)** were cultured in the presence of pooled HIV-1 peptides (5 µM) for 18 h to evaluate the number of IFN-γ-producing cells by ELISpot assay. SFU, spot forming units. Cutoff = 15 SFU/10^6^ cells and is represented by the dotted line. **(C,D)** Splenocytes were labeled with carboxyfluorescein succinimidyl ester (CFSE) (1.25 µM) and cultured in the presence of pooled HIV-1 peptides (5 µM) for 5 days to evaluate specific proliferation. After staining with fluorochrome-labeled anti-CD3, anti-CD4, and anti-CD8 monoclonal antibodies, cells were analyzed by flow cytometry. CFSE dilution on gated CD3^+^CD4^+^ **(C)** or CD3^+^CD8^+^ **(D)** cells was used as readout for antigen-specific proliferation. For detection of cytokine-producing T cells, cells were pulsed on day 4 for 12 h with pooled peptides in the presence of anti-CD28 and brefeldin A. Cells were then surface stained with anti-CD3, CD4, and CD8, permeabilized, and stained for intracellular cytokines. Multiparameter flow cytometry was used to determine the frequency of IFNγ-, IL2-, or TNFα-producing CD4^+^ and CD8^+^ T cells **(E,F)**. After gating on proliferating (CFSE^low^) and cytokine-producing cells, Boolean combinations were then created using FlowJo software to determine the frequency of each response based on all possible combinations of cytokine-producing CD4^+^ **(E)** and CD8^+^ **(F)** T cells. One million events were acquired in a live lymphocyte gate. The percent of proliferating CD4^+^ and CD8^+^ CFSE^low^ cells was determined in the CD3^+^ cell population. The percentage of proliferating and cytokine-producing T cells was calculated subtracting the values of stimulated from non-stimulated cultures. NS, not significant; *p < 0.05; **p < 0.01; ***p < 0.001. Data represent mean ± SD.

In an attempt to improve the cellular immune response induced by the DNA vaccination alone and compare it to the homologous immunization with αDECHIVBr8 or with the heterologous pVAXHIVBr8 (prime)/αDECHIVBr8 (boost), we decided to administer one additional dose of either αDECHIVBr8 or pVAXHIVBr8. In this way, the homologous immunization groups received three doses of either αDECHIVBr8 or pVAXHIVBr8, while the heterologous immunization group received one dose of pVAXHIVBr8 followed by two boosters of αDECHIVBr8. Control groups received three doses of either αDEC or pVAX (Figure 5A). To assess the magnitude of the immune response, we evaluated specific IFNγ-producing cells in splenocytes from immunized mice stimulated with pooled peptides (Figure 5B). Once again, we observed no difference between animals immunized with three doses of αDECHIVBr8 and animals immunized with pVAXHIVBr8 once/αDECHIVBr8 twice. By contrast, the group immunized with pVAXHIVBr8 thrice presented a lower number of specific IFNγ-producing cells. In addition, as observed with the administration of two doses, CD4^+^ and CD8^+^ T cells (Figures 5C,D, respectively) from mice immunized with three doses of αDECHIVBr8, and one dose pVAXHIVBr8 followed by two doses of αDECHIVBr8 displayed higher T cell proliferation against the pooled HIV-1 peptides than three doses of pVAXHIVBr8. By contrast, mice immunized with three doses of pVAXHIVBr8 continued to present a lower percentage of proliferation when compared to the two previous groups. αDEC and pVAX immunized mice presented negligible numbers of IFNγ-producing cells and T cell proliferation. Boolean combinations of proliferating and cytokine-positive populations indicated that homologous immunization with αDECHIVBr8 (3×) or with pVAXHIVBr8 (1×)/αDECHIVBr8 (2×) induced polyfunctional CD4^+^ T cells that proliferated and produced simultaneously IFNγ/TNFα, or IFNγ, or TNFα only (Figure 5E). Similar results were observed when CD8^+^ T cells were analyzed (Figure 5F). CD4^+^ and CD8^+^ T cells from mice immunized with the controls αDEC and pVAX displayed negligible percentages of specific proliferating/cytokine-producing T cells. Taken together, these results indicate that an additional dose of pVAXHIVBr8 does not improve the magnitude of the T cell responses over immunization with αDECHIVBr8 mAb.

### Immunization with Chimeric αDECHIVBr8 mAb Induces Broad T Cell Responses

**Figure 6:**
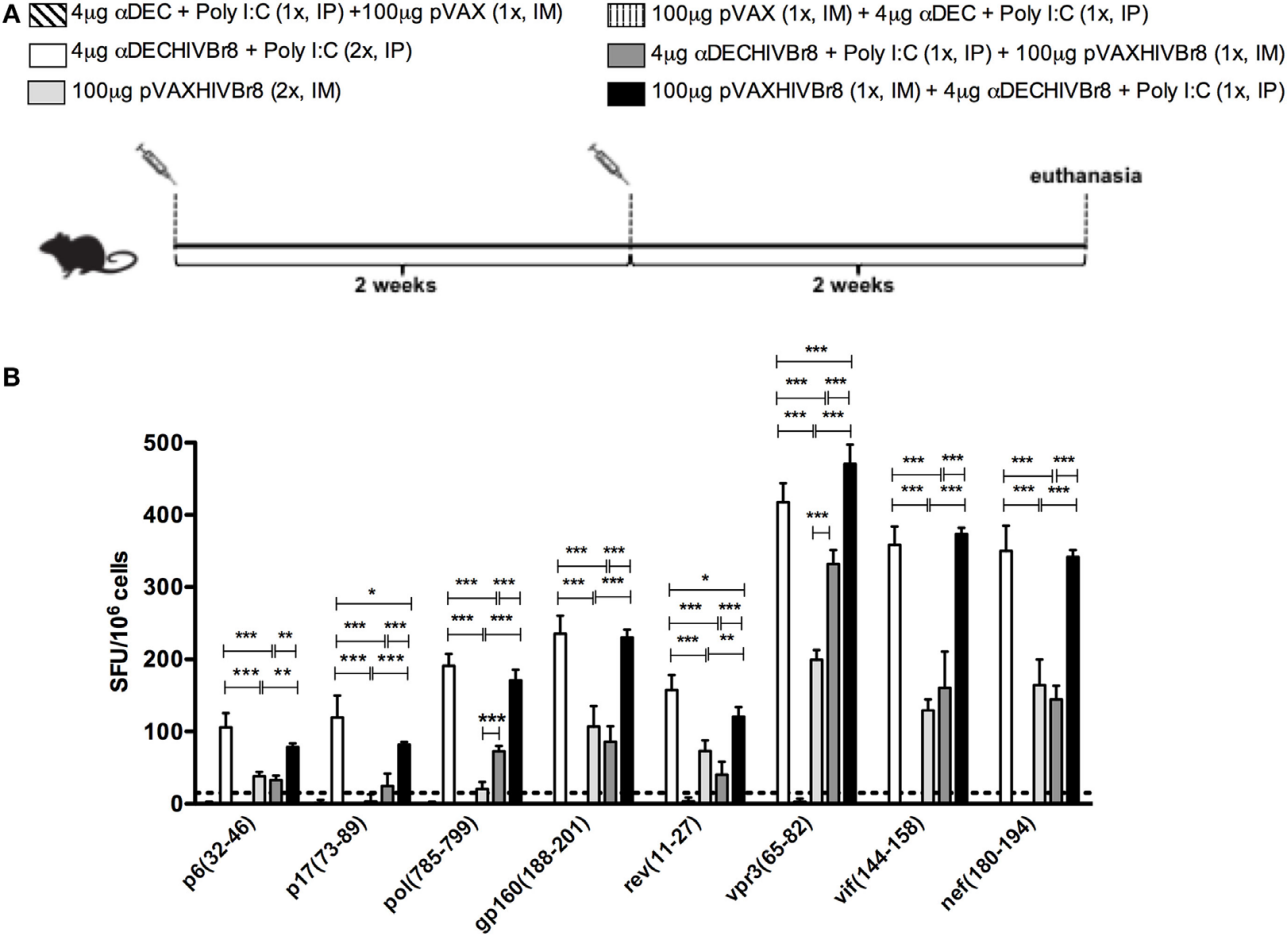
**Immunization with homologous or heterologous prime-boost induces broad T cell responses**. BALB/c mice (*n* = 6) were immunized with two doses of 4 µg of αDECHIVBr8 in the presence of poly (I:C) adjuvant (IP) or two doses of 100 µg of pVAXHIVBr8 DNA vaccine (IM). For heterologous regimens, mice were immunized with one dose of the DNA vaccine followed by one dose of monoclonal antibody. **(A)** Immunization scheme. Fifteen days after the last immunization, splenocytes were cultured **(B)** with individual HIV-1 peptides (5 µM) for 18 h to evaluate the number of IFN-γ-producing cells by ELISpot assay, SFU, spot forming units. Cutoff = 15 SFU/10^6^ cells and is represented by the dotted line.

To evaluate whether immunization induces broad T cell responses, splenocytes from mice immunized with two doses of αDECHIVBr8 and pVAXHIVBr8 or heterologous prime-boost regimens (Figure 6A) were incubated with each of the eight individual HIV-1 peptides present in the chimeric mAb or DNA vaccine. We detected IFNγ-producing cells against all tested peptides. Immunization with two doses of αDECHIVBr8 mAb or with pVAXHIVBr8 (prime)/αDECHIVBr8 (boost) elicited significantly higher numbers of IFNγ-producing cells when compared to mice immunized with two doses of pVAXHIVBr8 or that received αDECHIVBr8 (prime)/pVAXHIVBr8 (boost) (Figure 6B).

In summary, ours results demonstrate that immunization of BALB/c mice with two or three doses of αDECHIVBr8 mAb or heterologous prime-boost with pVAXHIVBr8 followed by αDECHIVBr8 were able to induce specific immune responses with a higher number of IFNγ-producing cells, and polyfunctional CD4^+^ and CD8^+^ T cells that proliferated and produced effector Th1 cytokines.

## Discussion

In this paper, we provide the first evidence that a multiepitopic vaccine can be targeted to DCs *via* DEC205 mAb. Furthermore, homologous prime-boosting with the αDECHIVBr8, or heterologous DNA prime followed by αDECHIVBr8 boosting encoding the same epitopes, can induce T cell responses of higher magnitude, polyfunctionality, and breadth than homologous prime-boost with the DNA vaccine.

In a previous study, our group demonstrated that a DNA vaccine encoding 18 HIV-derived promiscuous and preserved CD4^+^ T cell epitopes (16) was able to induce broad CD4^+^ and CD8^+^ T cell responses in different strains of HLA class II transgenic mice (DR2, DR4, DQ6, and DQ8) (18). Furthermore, the HIVBr18 DNA vaccine induced polyfunctional and long-lived CD4^+^ and CD8^+^ T cells responses to 8/18 HIV peptides encoded by the DNA vaccine (19). Despite promising, DNA vaccines show reduced immunogenicity in humans (21), and in the last decades, several strategies have been developed in order to increase the immunogenicity of these vaccines (17).

Initial attempts at developing a vaccine against HIV have focused mainly on the induction of humoral immune response against the viral gp120 protein and were not able to confer protection (44). In order to improve the induced immune response, the RV144 trial performed an immunization scheme that included a prime with a viral vector followed by a boost with Env protein and reported ~30% of protection in vaccinated individuals (1). A more detailed analysis of the immunized and protected individuals did not show CD8^+^ responses but CD4^+^ T cell responses to HIV correlated with reduced acquisition (2). Although the importance of CD8^+^ cytotoxic T cells as a first response against HIV (45, 46) is well established, a strong CD4^+^ T cell response is of utmost importance for the slow progression to AIDS (8). A recent study showed that untreated HIV-infected controllers presented higher Gag-specific CD4^+^ T cell responses and higher titers of nAbs against Env (10). Moreover, in non-human primates, depletion of CD4^+^ T cells markedly reduced protection mediated by vaccination after SIV challenge (47). Hence, an effective vaccine against HIV should induce specific cytotoxic responses as well as CD4^+^ T cell responses.

In an attempt to improve the HIV-specific cellular response, we targeted eight previously recognized epitopes derived from the HIVBr18 DNA vaccine directly to DCs. This is accomplished by the use of DC receptor-specific mAbs fused to the antigen of interest. We produced an αDEC205 chimeric mAb containing the sequence of eight HIV-derived CD4^+^ T cell epitopes (αDECHIVBr8) (18, 19) and compared DC targeting through αDECHIVBr8 mAb with the DNA vaccine pVAXHIVBr8. We initially showed that the αDECHIVBr8 mAb was successfully produced and retained its ability to bind to the DEC205 receptor, especially to the CD11c^+^CD8α^+^ DCs that naturally express the DEC205 receptor. The use of chimeric αDEC205 to deliver HIV antigens, especially Gag, to DCs has been previously reported (32, 33). Targeting Gag to CD8α^+^ DCs leads to a strong polyfunctional CD4^+^ (32, 37) and also CD8^+^ T cell responses (36) including in the gastrointestinal tract (34).

To address the minimum number of doses to induce specific immune responses, BALB/c mice were immunized with one or two doses of αDECHIVBr8 in the presence of poly (I:C) or with pVAXHIVBr8 DNA vaccine. We found that mice immunized with two doses of chimeric αDECHIVBr8 mAb developed a stronger CD4^+^ and CD8^+^ T cell response when compared to mice that received one dose of αDECHIVBr8 mAb or two doses of the DNA vaccine after stimulation with pooled HIV-1 peptides, demonstrating the effectiveness of the approach in the context of a chimeric multiepitope vaccine antigen. Next, we determined which immunization route was more effective. BALB/c received two doses of αDECHIVBr8 plus poly (I:C) by intraperitoneal (IP) or subcutaneous (SC) route. Although some studies have demonstrated the efficacy of the subcutaneous immunization with αDEC mAbs (27, 48), we detected a stronger CD4^+^ and CD8^+^ T cell response when αDECHIVBr8 was delivered IP. These results confirm what has already been demonstrated by other studies that used the IP route and showed high magnitude of specific cellular immune responses against different pathogens such as *Yersinia pestis* (49), *Plasmodium* sp. (28), papilloma virus (50), *Leishmania major* (51), Epstein–Barr virus (52), dengue virus (35), *T. cruzi* (30), and HIV (32, 33).

Some studies on the clinical course of HIV-1 have associated CD4^+^ T cells exhibiting polyfunctional profile with better control of disease. In elite controllers, superior polyfunctional CD4^+^ T cell response is observed when compared to non-controller individuals in HAART (53–55), including in the mucosal region (56). In RV144 trial analysis, vaccinees with polyfunctional CD4^+^ T cell responses against HIV peptides were found to have a lower rate of infection (57). Besides, the presence of specific HLA class II (DRB1*13, DQB1*06, and DRB1*15:02)-restricted CD4^+^ T cell responses (9, 14) play an important role in HIV immune control. In the present study, we show the induction of both polyfunctional CD4^+^ and CD8^+^ T cell responses after immunization with αDECHIVBr8 in the presence of poly (I:C).

When antigens are targeted to the DEC205^+^ DC population, they can be presented to CD4^+^ (58) as well as to CD8^+^ T cells (48). Other studies showed that antigens encapsulated in nanoparticles and targeted to the DEC205^+^ DCs are presented by MHC class I and induce specific CD8^+^ T cell responses (59, 60). This phenomenon can be explained by the ability of the CD11c^+^CD8α^+^ DCs to perform cross-presentation (61). Our study demonstrates that targeting promiscuous HIV epitopes to CD8α^+^ DC induce both CD4^+^ and CD8^+^ T cell-mediated immunity.

In recent years, several studies have shown that heterologous prime-boost immunization is able to increase the magnitude and quality of the immune response to many pathogens, including HIV (39). This approach started to be used in attempt to develop vaccines against pathogens that require more robust humoral and cellular immune responses such as HIV (62), *M. tuberculosis*, and *Plasmodium* sp. (42). In this work, we found that pVAXHIVBr8 DNA vaccine priming followed by boost with the αDECHIVBr8 mAb boosting increased the overall magnitude of the responses against HIV peptides when the opposite was tested (priming with αDECHIVBr8 followed by pVAXHIVBr8 boost). Our results have shown that receiving two doses of αDECHIVBr8 mAb or a pVAXHIVBr8/αDECHIVBr8 heterologous prime-boost induced a stronger CD4^+^ and CD8^+^ polyfunctional T cell response than any other immunization schemes tested. DNA vaccines have been shown to be most effective when administrated as a prime in vaccine formulations (63, 64). In our model, chimeric αDEC205 mAb was more effective as a boost exceptionally when the analysis was performed in non-proliferating (CFSE^high^) cytokine-producing cells. Of note, targeted αDEC205-Gag has been successfully used as a priming platform followed by recombinant vaccinia as boost to induce robust cellular immunity (34, 65).

In an attempt to improve the pVAXHIVBr8 magnitude, we immunized mice with three doses in the homologous or heterologous prime-boost regimens. We analyzed the ability of the cells from immunized mice to produce cytokines and proliferate at the same time. Overall, we observed the same phenomenon that occurred in animals that received only two doses showing that an additional dose of pVAXHIVBr8 did not increase the magnitude to the same level as immunization with αDECHIVBr8 (3×) or with pVAXHIVBr8 prime (1×)/αDECHIVBr8 (2×).

Our results have shown that immunization with two doses of αDECHIVBr8 mAb or with pVAXHIVBr8 followed by αDECHIVBr8 boost elicited higher magnitude T cell responses against all the peptides indicating a broad T cell response. Indeed, recent efficacy trials of T cell-based HIV vaccines showed that for protection, it is necessary to induce broad T cell responses toward conserved epitopes (2, 46, 66). An adenovirus-based SIV vaccine encoding eight virus proteins elicited broad T cell responses that reduced viremia after heterologous challenge (67) and the vaccine-induced response correlated with higher CD4^+^ T cell responses (68). Hence, vaccine candidates that expand the breadth of CD4^+^ and CD8^+^ T cell responses must be developed in order to provide optimal T-cell responses that may cope with the diverse circulating strains of HIV (69).

Our results provide evidence that multiepitope targeting to DCs can provide superior CD4^+^ and CD8^+^ T cell responses as compared to DNA vaccines alone. Given the known protective potential of CD4^+^ T responses elicited by αDEC205 mAb DC-targeted immunization (33), direct DC targeting, or the combination of DNA priming followed by DC targeting, is a promising platform for increasing HIV immunity after vaccination.

## Author Contributions

JA, SB, and DR conceived and designed the experiments. JA, VL, MY, HS, and DR performed the experiments. JA and DR analyzed the data and prepared the figures. DR, SB, and EC-N contributed with reagents and materials. JA, SB, and DR wrote the manuscript. SB, EC-N, and DR performed the final review of the article. All the authors read and approved the final article.

## Conflict of Interest Statement

The authors declare that the research was conducted in the absence of any commercial or financial relationships that could be construed as a potential conflict of interest.

## Supplementary Material

Figure S1 **Flow cytometer analysis of cellular proliferation and intracellular cytokine production**. Representative dot plots of a seven-color flow cytometry panel used for the detection of carboxyfluorescein succinimidyl ester (CFSE), IFNγ-, IL2-, and TNFα-producing T cells after *in vitro* stimulation with pooled HIV peptides. After gating on proliferating (CFSE^low^) or non-proliferating (CFSE^high^) and cytokine-producing cells, Boolean combinations were then created using FlowJo software to determine the frequency of each response based on all possible combinations of cytokine-producing T cells. The frequency of IFNγ-, IL2-, or TNFα cytokine-producing CFSE^low^ or CFSE^high^ CD4^+^ T cells is displayed.

Figure S2 **Homologous αDECHIVBr8 monoclonal antibody (mAb) immunization or heterologous prime-boost induces polyfunctional T cell responses**. BALB/c mice (*n* = 6) were immunized with two doses of 4 µg of αDECHIVBr8 in the presence of poly (I:C) adjuvant (IP) or two doses of 100 µg of pVAXHIVBr8 DNA vaccine (intramuscular). For heterologous regimens, mice were immunized with one dose of αDECHIVBr8 followed by one dose of DNA vaccine or *vice versa*. The control groups were immunized with one dose of αDEC mAb together with poly (I:C) followed by one dose with pVAX or *vice versa*. Fifteen days after the second dose, the spleen of each animal was removed and the splenocytes were labeled with carboxyfluorescein succinimidyl ester (CFSE) (1.25 µM) and cultured in the presence of pooled HIV-1 peptides (5 µM) for 5 days to evaluated specific proliferation. For detection of cytokine-producing T cells, cells were pulsed on day 4 for 12 h with pooled peptides in the presence of anti-CD28 and brefeldin A. Cells were then surface stained with anti-CD3, CD4, and CD8, permeabilized, and stained for intracellular cytokines. Multiparameter flow cytometry was used to determine the frequency of IFNγ-, IL2-, or TNFα-producing CD4^+^ and CD8^+^ T cells. After gating on non-proliferating (CFSE^high^) and cytokine-producing cells, Boolean combinations were then created using FlowJo software to determine the frequency of each response based on all possible combinations of cytokine-producing CD4^+^ **(A)** and CD8^+^ **(B)** T cells. One million events were acquired in a live lymphocyte gate. The percentage of proliferating and cytokine-producing T cells was calculated subtracting the values of stimulated from non-stimulated cultures. NS, not significant; **p* < 0.05; ***p* < 0.01; ****p* < 0.001. Data represent mean ± SD.
