# Supplementary figures and images for: Dendritic Cell Targeting Effectively Boosts T Cell Responses Elicited by an HIV Multiepitope DNA Vaccine
Source: Front Immunol. 2017 Feb 7;8:101. doi: 10.3389/fimmu.2017.00101 (PMC5295143; doi:10.3389/fimmu.2017.00101)

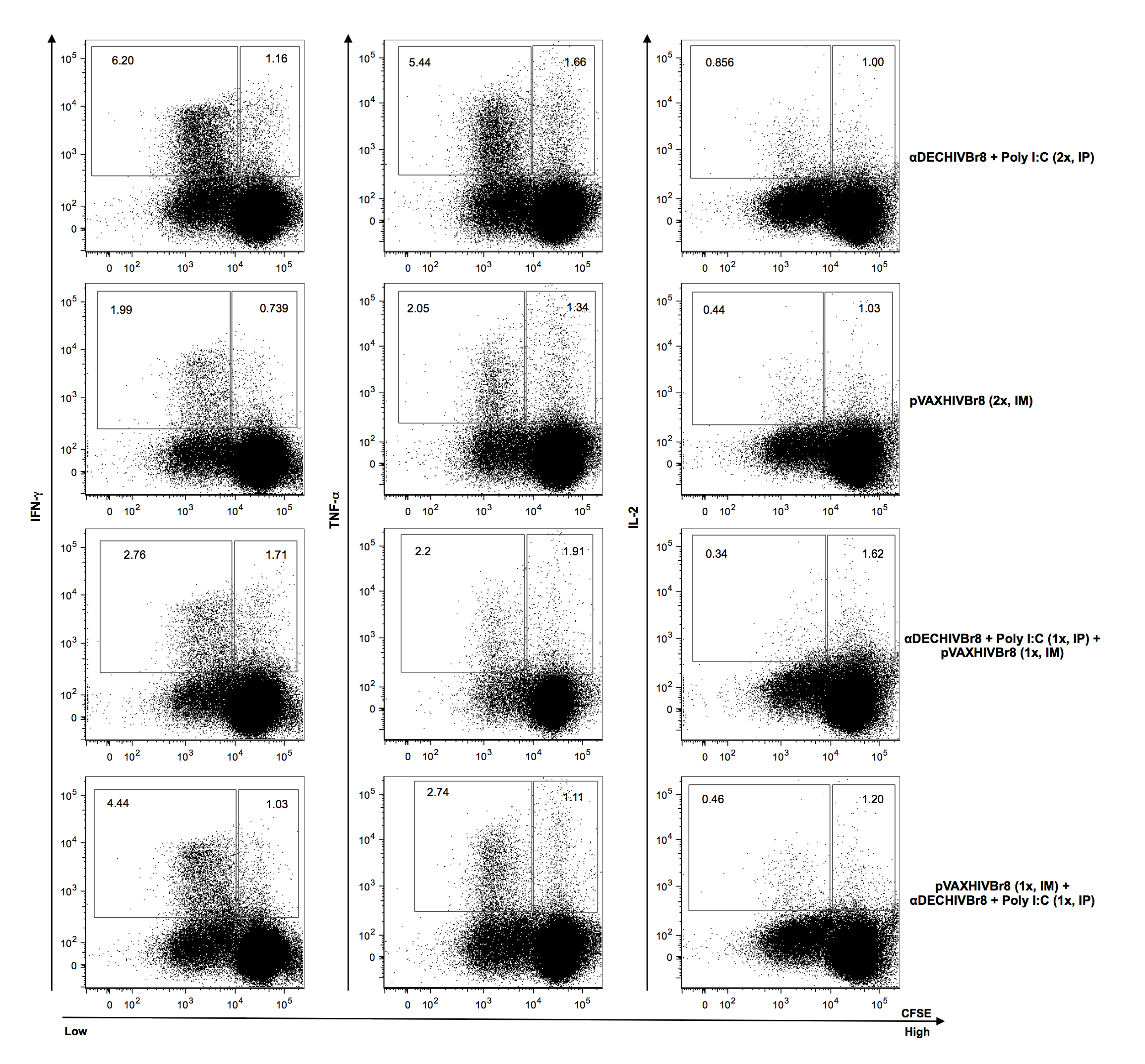

Supplement: Figure S1 — Flow cytometer analysis of cellular proliferation and intracellular cytokine production. Representative dot plots of a seven-color flow cytometry panel used for the detection of carboxyfluorescein succinimidyl ester (CFSE), IFNγ-, IL2-, and TNFα-producing T cells after in vitro stimulation with pooled HIV peptides. After gating on proliferating (CFSElow) or non-proliferating (CFSEhigh) and cytokine-producing cells, Boolean combinations were then created using FlowJo software to determine the frequency of each response based on all possible combinations of cytokine-producing T cells. The frequency of IFNγ-, IL2-, or TNFα cytokine-producing CFSElow or CFSEhigh CD4+ T cells is displayed. [file image_1.tiff]

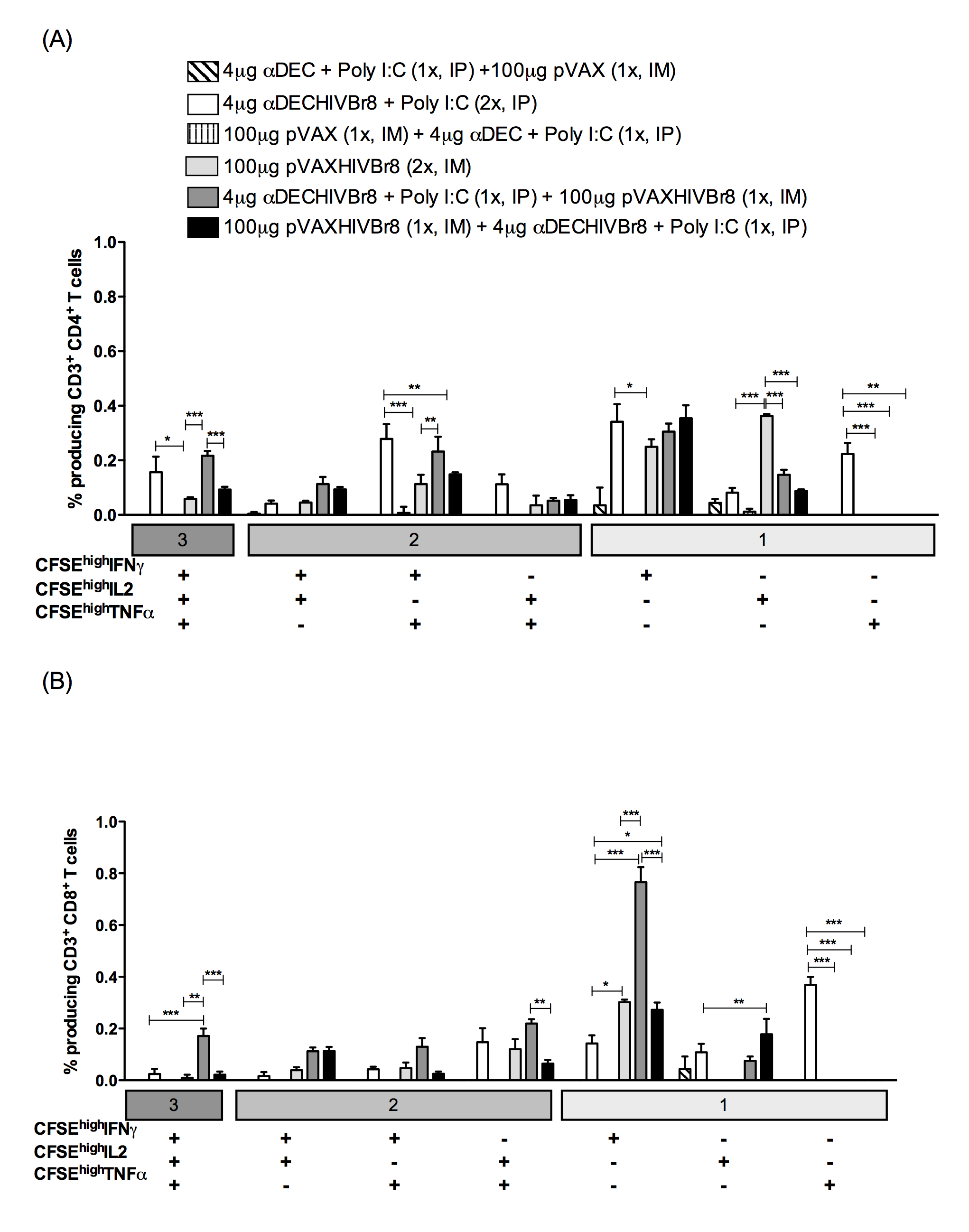

Supplement: Figure S2 — Homologous αDECHIVBr8 monoclonal antibody (mAb) immunization or heterologous prime-boost induces polyfunctional T cell responses. BALB/c mice (n = 6) were immunized with two doses of 4 µg of αDECHIVBr8 in the presence of poly (I:C) adjuvant (IP) or two doses of 100 µg of pVAXHIVBr8 DNA vaccine (intramuscular). For heterologous regimens, mice were immunized with one dose of αDECHIVBr8 followed by one dose of DNA vaccine or vice versa. The control groups were immunized with one dose of αDEC mAb together with poly (I:C) followed by one dose with pVAX or vice versa. Fifteen days after the second dose, the spleen of each animal was removed and the splenocytes were labeled with carboxyfluorescein succinimidyl ester (CFSE) (1.25 µM) and cultured in the presence of pooled HIV-1 peptides (5 µM) for 5 days to evaluated specific proliferation. For detection of cytokine-producing T cells, cells were pulsed on day 4 for 12 h with pooled peptides in the presence of anti-CD28 and brefeldin A. Cells were then surface stained with anti-CD3, CD4, and CD8, permeabilized, and stained for intracellular cytokines. Multiparameter flow cytometry was used to determine the frequency of IFNγ-, IL2-, or TNFα-producing CD4+ and CD8+ T cells. After gating on non-proliferating (CFSEhigh) and cytokine-producing cells, Boolean combinations were then created using FlowJo software to determine the frequency of each response based on all possible combinations of cytokine-producing CD4+ (A) and CD8+ (B) T cells. One million events were acquired in a live lymphocyte gate. The percentage of proliferating and cytokine-producing T cells was calculated subtracting the values of stimulated from non-stimulated cultures. NS, not significant; *p < 0.05; **p < 0.01; ***p < 0.001. Data represent mean ± SD. [file image_2.tiff]
